# Whole-genome sequencing of *Helicobacter pylori* isolates from Native American gastric biopsy specimens

**DOI:** 10.1128/mra.00160-24

**Published:** 2024-08-14

**Authors:** Kimberly Celona, Charles H. D. Williamson, Rishi Dholakia, Jason W. Sahl, Fernando P. Monroy, Erik W. Settles

**Affiliations:** 1 The Pathogen and Microbiome Institute, Northern Arizona University, Flagstaff, Arizona, USA; 2 Winslow Indian Health Care Center, Winslow, Arizona, USA; 3 Department of Biological Sciences, Northern Arizona University, Flagstaff, Arizona, USA; Loyola University Chicago, Chicago, Illinois, USA

**Keywords:** *Helicobacter pylori*, gastric cancer, gastrointestinal diseases, Navajo Nation, Native American

## Abstract

*Helicobacter pylori* infection has been linked to gastrointestinal diseases including gastric cancer. High rates of *H. pylori* infection and gastric cancer have been reported in indigenous populations within the United States. We report whole-genome sequencing of three *H*. *pylori* isolates originating from Native American patients presenting with gastric disease.

## ANNOUNCEMENT

*Helicobacter pylori*, a Gram-negative bacterium, is the causative agent of most human gastric infections and is linked to gastric cancer (1–3). Indigenous communities in the United States have elevated rates of infection and gastric cancer. Members of the Navajo Nation have rates of infection ranging between 56% and 70% (4, 5) and gastric cancer rates three to four times higher than non-Hispanic white populations (6). Navajo patients with gastric symptoms had a gastric biopsy during endoscopy and *H. pylori* culturing or PCR was performed. *H. pylori* was present in ~23% of these biopsy samples (7). We describe whole-genome sequencing of three *H*. *pylori* isolates from these patients.

Patients were enrolled by informed consent (Navajo Nation IRB #NNR16.263). Gastric pinch biopsy samples were collected during a routine scheduled patient endoscopy. Samples were ground in sterile phosphate-buffered saline, inoculated onto Columbia Agar plates containing 5% defibrinated sheep blood and *H. pylori* selective supplement (Dent), and incubated for 72 h at 37°C under microaerophilic conditions (5% O_2_, 10% CO_2_, and 85% N_2_). Minimum inhibitory concentrations (MICs) for clarithromycin and metronidazole were determined with ETESTs (bioMérieux) by inoculating 100 µL of a 3 McFarland equivalent isolate suspension onto Mueller-Hinton II plates with 5% defibrinated sheep blood and incubating at 37°C for 4 days under microaerophilic conditions.

Isolate genomic DNA was extracted using a Blood and Tissue Kit (Qiagen) following the manufacturer’s protocol with the additional pretreatment for Gram-negative bacteria. Whole-genome sequencing libraries were generated as previously described (8, 9) except quality was assessed with a Fragment Analyzer using the High Sensitivity NGS fragment kit. Samples were sequenced on the MiSeq platform. Contaminating sequencing reads were identified and removed with the BBsplit tool (BBMap v38.93—sourceforge.net/projects/bbmap/) using phiX (J02482.1) and human (GCF_000001405.39) genomes as references, followed by assignment of taxonomic classifications to reads with kraken2 v2.1.2 (10) and removal of contaminating reads. *H. pylori* genomes were assembled using SPAdes v3.15.3 (--careful, --cov-cutoff auto) (11). Depth of coverage was calculated from minimap2 v2.24 (-ax sr) (12) alignments using Samtools v1.16.1 (13). Contigs with anomalously low depth of coverage were removed. Assembly metrics were calculated with the statswrapper.sh tool (sourceforge.net/projects/bbmap/ v39.01). Assemblies were annotated with the NCBI Prokaryotic Genome Annotation Pipeline (PGAP) v6.6 (14). Core genome single nucleotide polymorphisms (SNPs) were called from nucmer v3.1 (15) alignments (reference = GCA_017821535.1) within NASP v1.2.1 (16), and a phylogeny was inferred with IQ-TREE v2.2.2.3 (17, 18) from proximity filtered SNPs (distance of 5). The *vacA* and *cagA* genotypes were determined with *in silico* PCR (usearch v11.0.667_i86linux32 – search_pcr, -maxdiffs 2) (19) using previously described primers (20–23). Genomes were screened for antibiotic-resistance markers listed in the Comprehensive Antimicrobial Resistance Database (24).

**Fig 1:**
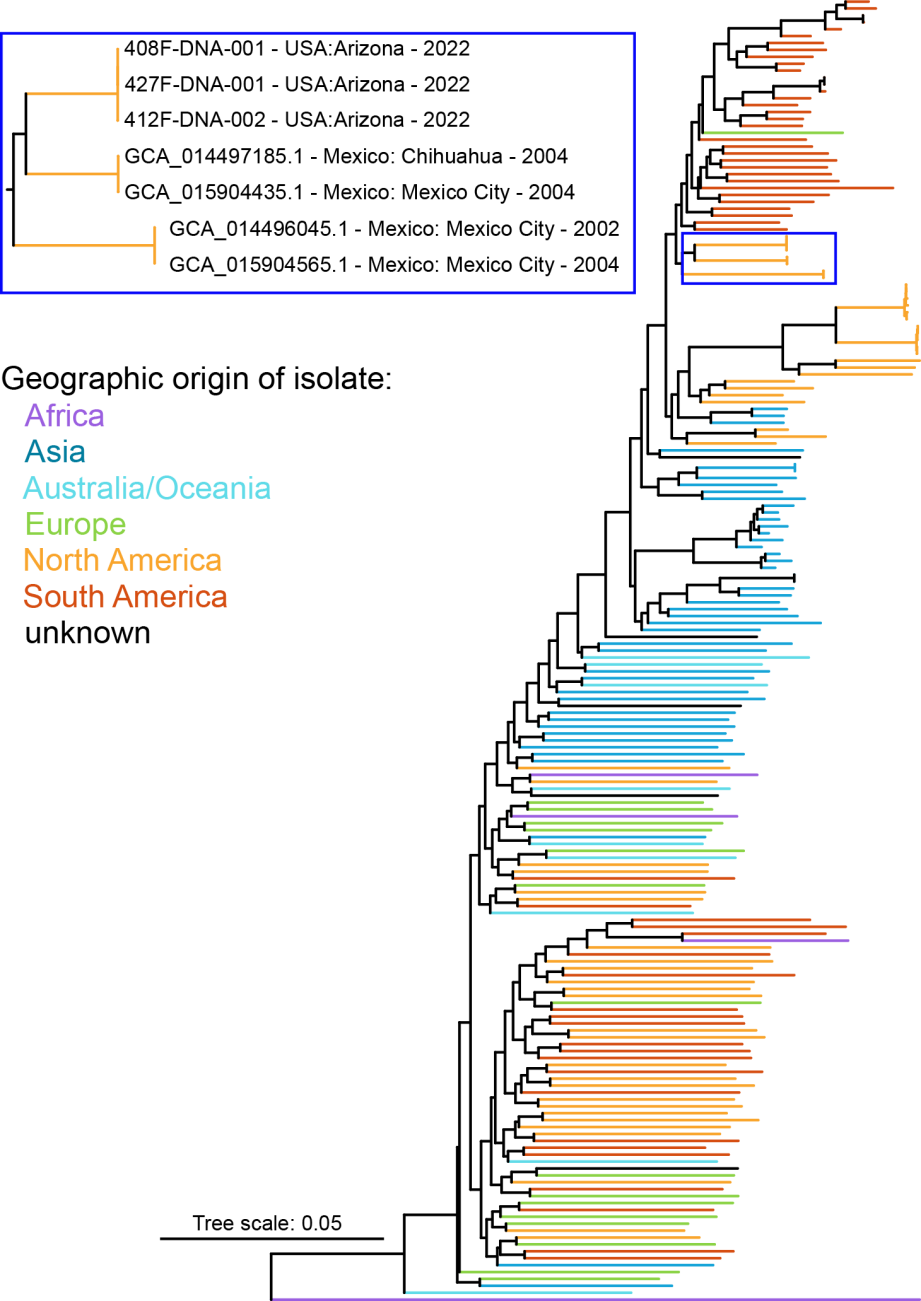
Core genome SNP phylogeny (midpoint rooted) of 186 publicly available *H. pylori* genomes and three newly sequenced *H. pylori* genomes. Colors indicate the continent of origin for the *H. pylori* isolates included in the tree. The blue box highlights the three newly sequenced isolates (408F, 412F, and 427F) and closely related isolates. The three newly sequenced isolates are closely related to isolates collected from Indigenous or Mestizo individuals presenting with gastritis in Mexico.

Genome assembly information is presented in Table 1. The three isolates are putatively genotyped as *cagA*^−^ and *vacA* type s2i2m2. A SNP phylogeny (Fig. 1) indicates that the isolates are closely related to isolates originating from Indigenous or Mestizo individuals presenting with gastritis in Mexico (25, 26). ETESTS indicate some isolates are resistant to clarithromycin and metronidazole (Table 1).

**TABLE 1:** Genome assembly metrics and accession numbers

Isolate	408F-DNA-001	412F-DNA-002	427F-DNA-001
Assembly accession	JAYXIY000000000	JAYXIX000000000	JAYXIW000000000
SRA accession	SRR27606653	SRR27606652	SRR27606651
Sequencing kit	500-cycle Nano v2	500-cycle Nano v2	600-cycle v3
Sequencing format	2 × 251 bp	2 × 251 bp^*a*^	2 × 301 bp
Total number of paired reads	373,565	441,603	1,201,328
Average depth of coverage	113×	124×	436×
Number of contigs	28	19	18
Genome size (bp)	1,570,731	1,570,272	1,570,218
L50	4	3	3
N50 (bp)	176,074	236,910	236,904
Length of longest contig (bp)	266,682	411,760	411,746
Average GC content	0.39	0.39	0.39
Total CDSs (PGAP)	1,502	1,487	1,490
Minimum inhibitory concentration for clarithromycin (*R* > 0.25 µg/mL)^*b*^	0.125	1.5	0.5
Minimum inhibitory concentration for metronidazole (*R* > 8 µg/mL)^*c*^	8	16	1
Mutations potentially associated with clarithromycin resistance^*d*^	Mutations within 23S rRNA (ARO:3004134) - T510C, G722A, G760del, T896C, T976G, T1024C, C1516del, T1568C, C1648T, T2199C		
Genes/mutations potentially associated with metronidazole resistance^*e*^	Presence of major facilitator superfamily antibiotic efflux pump (ARO:3003964); mutations within frxA (ARO:3007059) - **V7I**, **A16T**, Q27E, I44V, L71I, F72S, G73S, T110A, N111D, N124S, M126I, A154V, E176K, C193S; mutations within rdxA (ARO:3007055) - **T31E**, **D59N**, L62V, S88P, G98S, **A118S**, V123T, R131K, E175Q		

^*A*^ Reverse reads trimmed to 228 nucleotides due to sequencing quality.

^*b*^ Resistance breakpoint for clarithromycin—EUCAST v13.1.

^*c*^ Resistance breakbpoint for metronidazole—EUCAST v13.1.

^*d*^ Genomic data queried against features associated with clarithromycin resistance in *H. pylori* in the Comprehensive Antimicrobial Resistance Database. The mutations in Table 1 were identified within the 23S rRNA gene for all three isolates, but specific mutations listed within the CARD were not identified.

^*e*^ Genomic data queried against features associated with metronidazole resistance in *H. pylori* in the Comprehensive Antimicrobial Resistance Database. An antibiotic efflux pump gene was identified in all three genomes. Mutations were identified within frxA and rdxA in all three genomes; specific mutations listed within the CARD are in bold text.

## Data Availability

The whole-genome sequencing project has been deposited under NCBI BioProject PRJNA1066305. Assembly accession numbers and Sequence Read Archive accession numbers are included in Table 1.
